# Supplementary material for: The Introduction of Detergents in Thermal Proteome Profiling Requires Lowering the Applied Temperatures for Efficient Target Protein Identification
Source: Molecules. 2023 Jun 20;28(12):4859. doi: 10.3390/molecules28124859 (PMC10301966; doi:10.3390/molecules28124859)
Supplement: Supplementary file 1 [file molecules-28-04859-s001.zip › Supplementary_Figure_S1-S3.pdf]

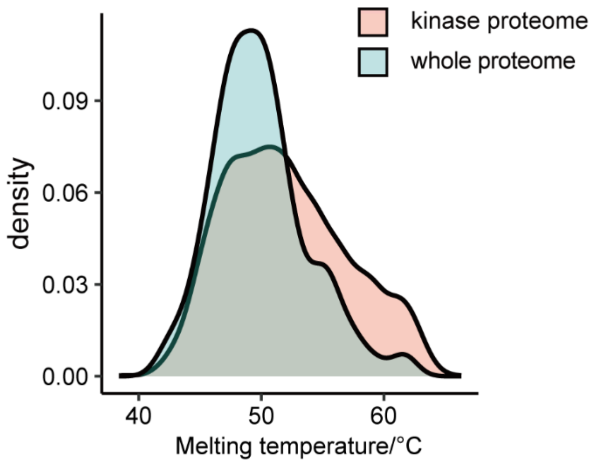

Figure S1. Melting temperature distributions of the kinase proteome and the whole proteome.

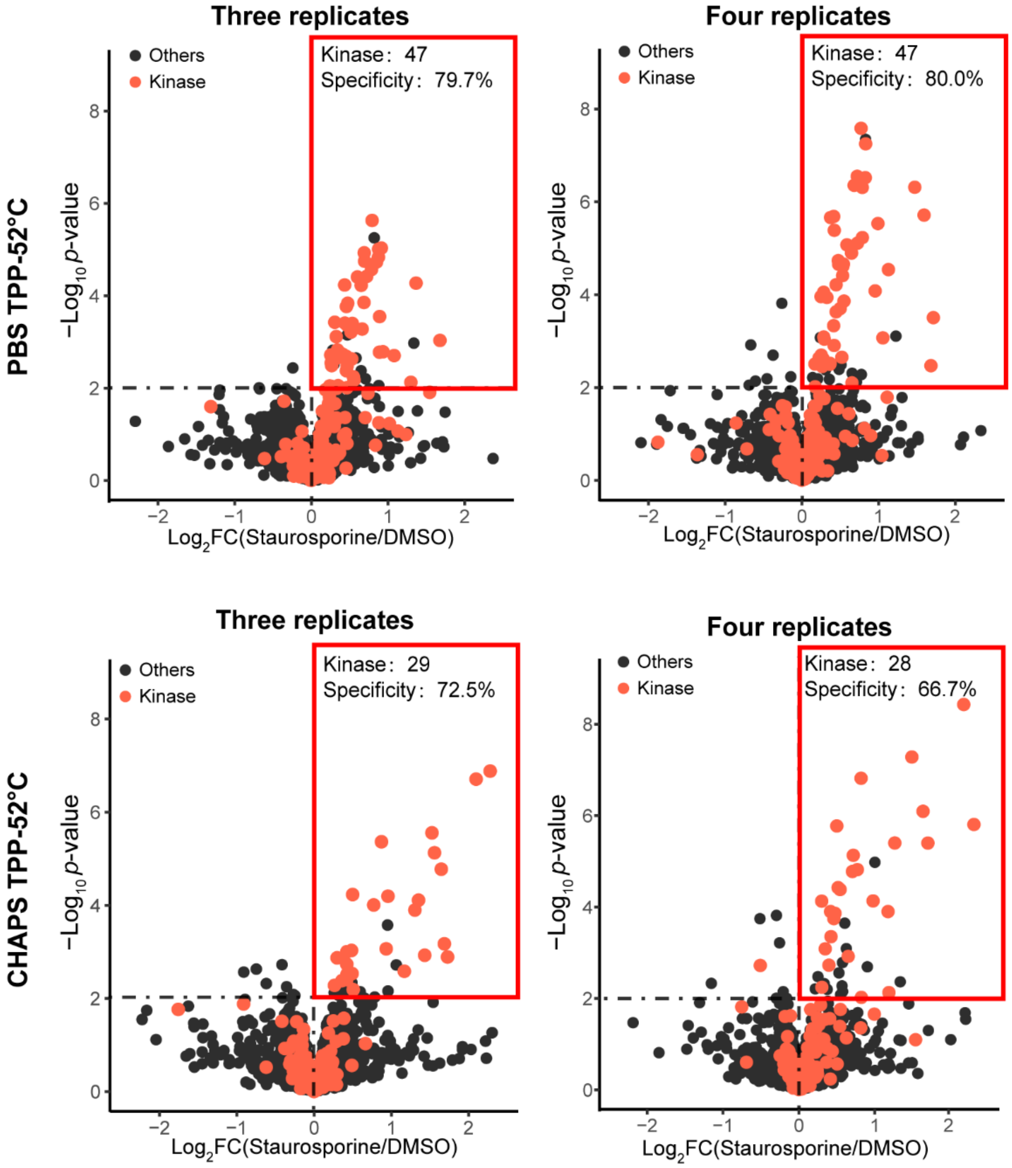

Figure S2. Comparing target identification performance of TPP between performing

three replicates and four replicates. **(A)** Volcano plot visualization of staurosporine kinase targets identified by PBS-TPP at 52 °C with three replicates (left) and with four replicates (right). **(B)** Volcano plot visualization of staurosporine kinase targets identified by CHAPS-TPP at 52 °C with three replicates (left) and with four replicates (right).

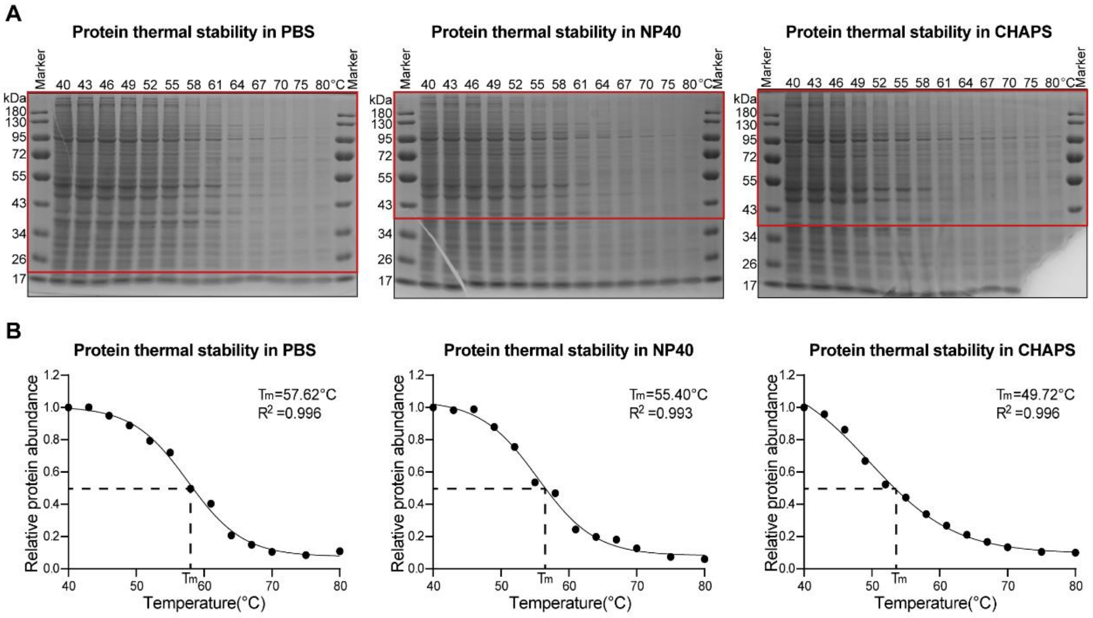

Figure S3. Comparing the thermal stabilities of the proteome in PBS, in PBS with 0.4% NP40, and in PBS with 1% CHAPS. (**A**) SDS-PAGE readout of proteins that were collected from the supernatants after heating. From left to right: in the context of PBS (**left**), in the context of PBS supplemented with 0.4% NP40 (**middle**), and in the context of PBS supplemented with 1% CHAPS (**right**). (**B**) Normalized protein quantification value according to the area denoted by the red frame in (A). Tm represents melting temperature; R^2 refers to the Pearson correlation between the actual values and the fitted values of the four-parameter logistic curve. The Tm curves were fitted using Prism (version 8.0.2).
